# Serum histone H3 levels and platelet counts are potential markers for coagulopathy with high risk of death in septic patients: a single-center observational study

**DOI:** 10.1186/s40560-019-0420-2

**Published:** 2019-12-26

**Authors:** Takashi Ito, Takaaki Totoki, Yayoi Yokoyama, Tomotsugu Yasuda, Hiroaki Furubeppu, Shingo Yamada, Ikuro Maruyama, Yasuyuki Kakihana

**Affiliations:** 1 Department of Emergency and Intensive Care Medicine, Kagoshima University Graduate School of Medical and Dental Sciences, Kagoshima, Japan; 2 Department of Systems Biology in Thromboregulation, Kagoshima University Graduate School of Medical and Dental Sciences, Kagoshima, Japan; 3 R&D Center, Shino-Test Corporation, Sagamihara, Japan

**Keywords:** Sepsis, Disseminated Intravascular coagulation (DIC), Diagnosis, Histone

## Abstract

**Background:**

Recent studies have suggested that anticoagulant therapy does not confer a survival benefit overall in sepsis, but might be beneficial in sepsis-associated disseminated intravascular coagulation (DIC). In particular, those with high Sequential Organ Failure Assessment (SOFA) scores might be the optimal target for anticoagulant therapy. However, both DIC and SOFA scores require the measurement of multiple markers. The purpose of this study was to explore a minimal marker set for determining coagulopathy at high risk of death in septic patients, wherein histone H3 levels were evaluated as indicators of both organ failure and coagulation activation.

**Methods:**

We analyzed correlations among levels of serum histone H3 and other coagulation markers in 85 cases of sepsis using Spearman’s rank correlation test. We then compared the utility of histone H3 to that of other coagulation markers in predicting the traditional DIC state or 28-day mortality by receiver-operating characteristics analysis. Finally, we suggested cut-off values for determining coagulopathy with high risk of death, and evaluated their prognostic utility.

**Results:**

Serum histone H3 levels significantly correlated with thrombin-antithrombin complex (TAT) levels (Spearman’s ρ = 0.46, *p* < 0.001), and weakly correlated with platelet counts (Spearman’s ρ = − 0.26, *p* < 0.05). Compared to other coagulation markers, histone H3 levels showed better performance in predicting 28-day mortality. When combining serum histone H3 levels with platelet counts, our new scoring system showed a concordance rate of 69% with the traditional four-factor criteria of DIC established by the Japanese Association for Acute Medicine. The 28-day mortality rates of the new and the traditional criteria-positive patients were 43% and 21%, respectively. Those of the new and the traditional criteria-negative patients were 5.7% and 9.4%, respectively.

**Conclusions:**

Serum histone H3 levels and platelet counts are potential markers for determining coagulopathy with high risk of death in septic patients. Further studies are needed to clarify the utility of serum histone H3 levels in the diagnostic of coagulopathy/DIC.

## Background

Sepsis is a life-threatening disorder resulting from dysregulated host responses to infection [1]. Although the details of dysregulated host responses are not defined, insufficiency of microcirculation associated with excessive activation of the complement system, the coagulation system, and the innate immune system might be involved [2]. These exaggerated proinflammatory and procoagulant responses have been the predominant target of clinical intervention; however, none has proven effective to date [3]. Considering that sepsis is a particularly heterogeneous syndrome, the selection of patients who may benefit from a certain intervention is required [2].

Recent studies have suggested that anticoagulation therapy does not confer a survival benefit overall in sepsis, but might be beneficial in the specific subpopulation with sepsis-associated disseminated intravascular coagulation (DIC) [4–6], with those with high disease severity as the optimal target for anticoagulation [7]. Several organizations have proposed diagnostic criteria for DIC, including the International Society on Thrombosis and Haemostasis (ISTH) [8], the Japanese Association for Acute Medicine (JAAM) [9], and the Japanese Society on Thrombosis and Hemostasis (JSTH) [10]. However, DIC is not routinely diagnosed in most institutions in part because diagnostic criteria require the measurement of multiple coagulation markers, such as prothrombin time (PT), fibrin(ogen) degradation products (FDP), and thrombin-antithrombin complex (TAT), some of which are not commonly available.

Histone H3 is one of the nucleosome core proteins, acting as a spool around which DNA winds. In septic conditions, histone H3 and other histones can be released from activated neutrophils and severely injured cells into the extracellular space, where they induce platelet aggregation [11, 12], coagulation activation [13], endothelial damage [14, 15], and cardiac injury [16]. Recently, we developed an enzyme-linked immunosorbent assay (ELISA) which can measure serum or plasma histone H3 levels with high sensitivity and specificity [17]. Using this ELISA, we found that circulating histone H3 levels in septic patients were associated with coagulopathy, multiple organ failure, and death [18]. These findings led us to speculate that serum histone H3 levels might be a simple biomarker for selecting coagulopathy patients at high risk of death who may benefit from anticoagulation therapy [5, 7]. In this study, we explore a minimal marker set for determining coagulopathy at high risk of death in septic patients, wherein histone H3 levels were evaluated as indicators of both organ failure and coagulation activation.

## Methods

### Study population

This investigation was performed using a data set from a single-center observational study conducted in Kagoshima University Hospital between July 2015 and June 2016 [18]. Among 85 cases of sepsis enrolled in this study, serum samples were obtained in 81 patients within 24 hours after intensive care unit admission (ICU day 1) and analyzed for histone H3 levels. Diagnosis of DIC was made according to the JAAM DIC criteria on day 1, 3, 5, and 7. The Acute Physiology and Chronic Health Evaluation II (APACHE II) scores and the Sequential Organ Failure Assessment (SOFA) scores were calculated with reference to the medical records although the APACHE II scores and the SOFA scores in this study did not include the Glasgow Coma Scale scores due to invalid assessment of the scores under sedation. The study was compliant with the Declaration of Helsinki and approved by the Ethics Committee of Kagoshima University.

### Measurement of histone H3 and coagulation markers

Serum histone H3 levels were measured by ELISA using antibodies against histone H3 (Shino-Test Corporation, Sagamihara, Japan) as described previously [17, 18]. The lower detection limit of the ELISA was 2 ng/mL, and the linearity was observed in the range up to 250 ng/mL. The ELISA specifically detected histone H3 and did not react with other histones, including histone H2A, H2B, and H4 [17, 18].

Plasma FDP and TAT levels were determined by LPIA FDP (LSI Medience, Tokyo, Japan) and STACIA CLEIA TAT (LSI Medience, Tokyo, Japan), respectively, according to the manufacturer’s instructions. Plasma PT and antithrombin (AT) activity were analyzed using HemosIL RecombiPlasTin (Instrumentation Laboratory Company, Bedford, MA, USA) and HemosIL Liquid Antithrombin (Instrumentation Laboratory Company), respectively, according to the manufacturer’s instructions. Platelet counts were analyzed using an automated counting device XE-5000 (Sysmex Corporation, Kobe, Japan).

### Statistical analysis

Statistical analysis was performed using commercial software (SPSS version 23 IBM, Inc., Armonk NY, USA). The relationship between serum histone H3 levels and other coagulation markers was analyzed by Spearman’s rank correlation test. The predictive ability of each parameter for the traditional DIC state established by JAAM or 28-day mortality was compared by receiver-operating characteristics (ROC) analysis. A *p* < 0.05 was considered statistically significant.

## Results

**Fig. 1:**
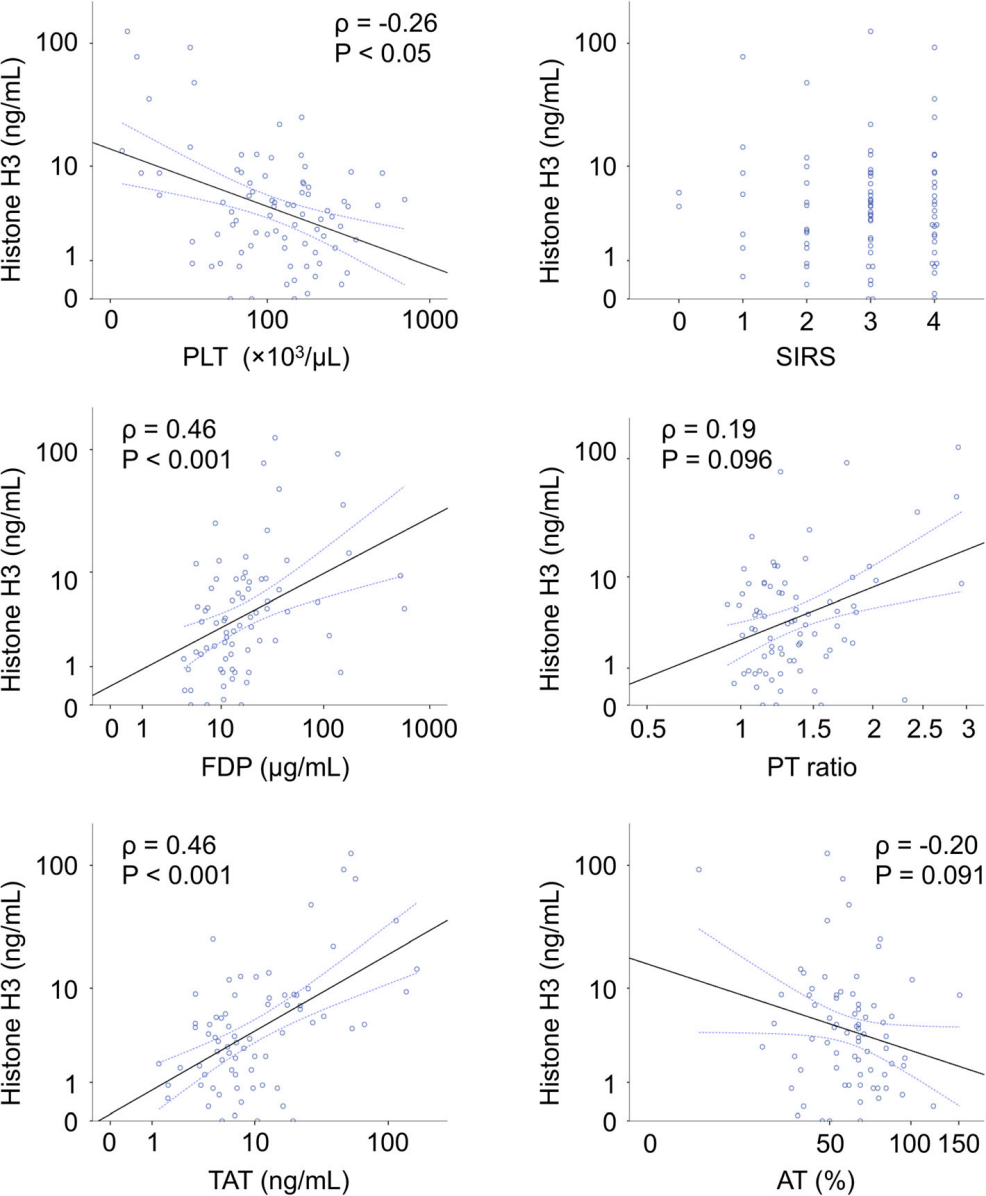
Serum histone H3 levels are correlated with coagulation markers. Correlations between serum histone H3 levels (ng/mL) and coagulation marker levels, including platelet counts (PLT, × 10^3^/μL), fibrin(ogen) degradation products levels (FDP, μg/mL), thrombin-antithrombin complex levels (TAT, ng/mL), SIRS score, prothrombin time (PT) ratio, and antithrombin activity (AT, %), on ICU day 1 are shown. Spearman’s ρ and *p* values, are also shown. Logarithmic scales are used, except for SIRS score.

First, we assessed the characteristics of histone H3 levels in the setting of sepsis-associated DIC by analyzing its correlation with other coagulation markers. The basic characteristics of the study population were shown previously [18]. As shown in Fig. 1, serum histone H3 levels were significantly correlated with TAT levels (Spearman’s ρ = 0.46, *p* < 0.001) and FDP levels (Spearman’s ρ = 0.46, *p* < 0.001), and weakly correlated with platelet counts (Spearman’s ρ = − 0.26, *p* < 0.05).

We then compared the diagnostic utility of histone H3 to that of other coagulation markers for predicting the traditional DIC state or 28-day mortality. The area under the ROC curve (AUC) of serum histone H3 levels for predicting the traditional DIC state was 0.75, approximately equivalent to the value of constituent makers for DIC, such as platelets (0.79), PT (0.60), FDP (0.76), and TAT (0.70), and better than that of high mobility group box 1, the prototypical damage-associated molecular pattern molecule. The AUC of serum histone H3 levels for predicting 28-day mortality was 0.73, better than that of other coagulation markers, such as platelets (0.72), PT (0.68), FDP (0.57), and TAT (0.58).

**Fig. 2:**
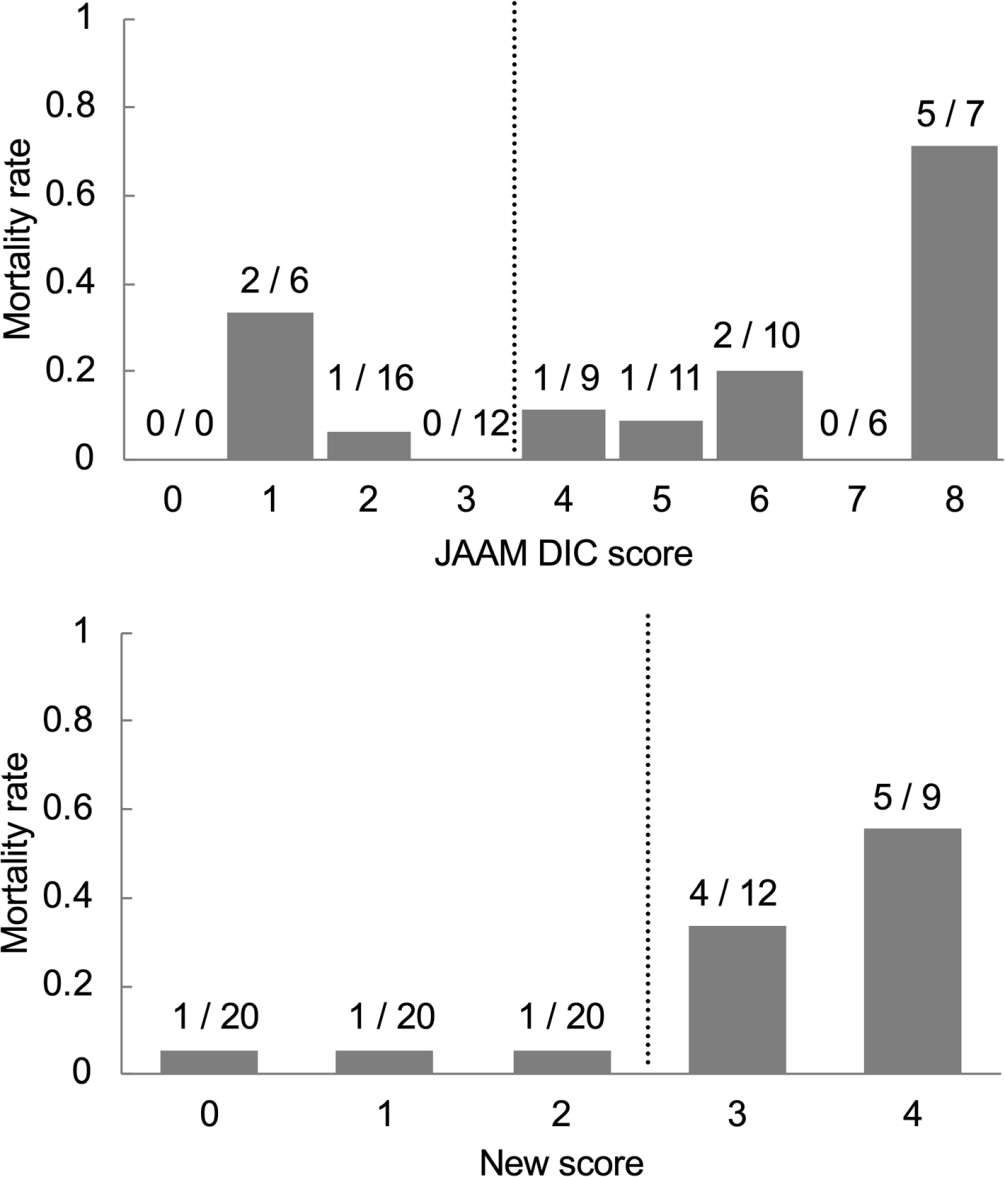
The new scoring system clearly detect patients at high risk of death. Mortality rates of patients with each JAAM DIC score and the new score on ICU day 1 are shown. Dotted lines represent cut-off lines for JAAM DIC criteria and the new scoring system. The numerator and denominator of a fraction represent the number of non-survivors divided by the sum of the survivors + non-survivors in each score.

Subsequently, we assessed whether serum histone H3 levels could be a good substitute for FDP, PT, and the disease severity markers for selecting coagulopathy patients at high risk of death. To this end, we proposed a new scoring system, consisting of platelet counts and serum histone H3 levels (Table 1). The cut-off values of serum histone H3 levels and platelet counts were determined by ROC analysis (Additional file 1: Figure S1). A sum score ≥ 3 was considered to be coagulopathy with high risk of death in the new scoring system. As shown in Table 2, the diagnostic concordance rate was 69% (51/74) between the traditional JAAM criteria and the new two-factor scoring system. The 28-day mortality rates of the new and traditional criteria-positive patients were 43% and 21%, respectively. The 28-day mortality rates of the new and traditional criteria-negative patients were 5.7% and 9.4%, respectively. Consistent with these findings, the new scoring system could clearly discriminate patients at high risk of death from those at low risk (Fig. 2). The AUC of the new scoring system for predicting 28-day mortality was 0.80, better than that of other prognostic scores in the ICU setting, such as the SOFA score (0.78) and the APACHE II score (0.71). Thus, the new scoring system could determine coagulopathy at high risk of death in septic patients in a simple manner.

**Table 1:** The new scoring system for identifying coagulopathy patients at high risk of death. The scoring system using serum histone H3 level and platelet counts is shown. A sum score of ≥ 3 is considered to be coagulopathy with high risk of death

Histone H3 (ng/mL)	
< 3	0
3 ≤ < 9	1
9 ≤	2
Platelet (× 10^3^/μL)	
> 120	0
80 ≤ < 120	1
≤ 80	2
Sum score	
3 ≤	Coagulopathy with high risk of death

**Table 2:** Diagnostic concordance rates between the JAAM DIC criteria and new scoring system. The diagnostic concordance rate between the JAAM DIC criteria and the new scoring system on ICU day 1 is 69%. This can be calculated by dividing the numbers of double-negative patients and double-positive patients (31 + 20) by the total number of patients (74). The mortality rates of the new and the JAAM DIC criteria-positive patients were 43% (9/21) and 21% (9/42), respectively. The mortality rates of the new and the JAAM DIC criteria-negative patients were 5.7% (3/53) and 9.4% (3/32), respectively

Number of patients		JAAM DIC score		
		< 4 (negative)	4 ≤ (positive)	Total
New score	< 3 (negative)	31	22	53
	3 ≤ (positive)	1	20	21
	total	32	42	74
Mortality rate		JAAM DIC score		
		< 4 (negative)	4 ≤ (positive)	Total
New score	< 3 (negative)	6.5% (2/31)	4.5% (1/22)	5.7% (3/53)
	3 ≤ (positive)	100% (1/1)	40% (8/20)	43% (9/21)
	Total	9.4% (3/32)	21% (9/42)	16% (12/74)

## Discussion

In this study, we propose a simple scoring system for determining coagulopathy at high risk of death in septic patients. The new scoring system is designed to be (i) simple, (ii) concordant with traditional DIC diagnosis, and (iii) prognostically relevant. A set of two parameters, platelet counts and serum histone H3 levels, showed excellent performance for this purpose.

Recent studies have suggested that the optimal target for anticoagulation therapy in septic conditions might be DIC patients with high disease severity [5, 7]. The JAAM DIC criteria are widely used in Japan and are recommended for the diagnosis of sepsis-associated DIC [19]. They consist of four parameters, namely, platelet count, FDP, PT, and systemic inflammatory response syndrome (SIRS) [9]. Allowing that no single biomarker has yet been reported by which DIC can be specifically diagnosed, combinational evaluation of four parameters might become a bottleneck in the diagnosis process. In the setting of sepsis, a decrease in platelet count is associated with impairment of vascular integrity [20]. An elevation of FDP is associated with activation of the coagulation and fibrinolysis pathways [21]. A prolongation of PT is generally associated with liver dysfunction, including shock liver, in the setting of sepsis [22]. SIRS is associated with an increased risk of coagulopathy and organ dysfunction [23]. Overall disease severity can be assessed by the SOFA score [5, 7, 24]. Circulating histone H3 levels are associated with multiple organ failure and coagulopathy [18] and significantly correlated with FDP levels (Fig. 1). Thus, circulating histone H3 levels can be a good substitute for FDP, PT, SIRS, and the disease severity marker. Indeed, when combining circulating histone H3 levels with platelet counts, our simple two-factor scoring system could identify 70% of patients with DIC and high disease severity and 83% of those without (Additional file 1: Table S1). Furthermore, with respect to the remaining 20% unmatched cohort, our two-factor scoring system showed good prognostic performance. These findings indicate that the new scoring system could identify coagulopathy patients with high disease severity in a simple manner.

The present study has several limitations. First, this is a retrospective observational study, and the cut-off values of our new scoring system were set to optimize diagnostic performance. Thus, the utility of the new scoring system could not be validated in this study and should be validated in future studies. Second, most of the JAAM DIC-positive and new scoring system-negative patients survived 28 days, but it is not clear whether anticoagulant therapy was dispensable in these patients. Anticoagulant therapy was conducted in 52% of enrolled patients based on the JAAM DIC scores and clinical features, and might have contributed to the survival of the JAAM DIC-positive and new scoring system-negative patients. Prospective intervention studies are needed for rigorous comparison of JAAM DIC criteria and our new scoring system. Third, DIC scores other than the JAAM DIC score, such as the ISTH DIC score, were not available in this study because of the retrospective nature. Fourth, applicability of the new scoring system to patients with hematologic disorders is not clear because thrombocytopenia can be observed in these patients independently of DIC and circulating histone H3 levels may not be elevated in leukopenic patients [17]. Fifth, histone H3 levels can be measured at present for research purposes only and cannot be measured in clinical settings. This is closely related to the novelty of this study, but is simultaneously related to the disadvantage. Further studies are required for the clinical application of histone H3 measurement. Finally, it is not clear in this study whether the elevation of histone H3 levels is a cause or consequence of DIC, although some studies have demonstrated that extracellular histones activate platelets and coagulation pathways [11–13, 25]. This may be important when considering a therapeutic strategy for DIC.

## Conclusions

Serum histone H3 levels correlated with FDP and TAT levels in septic patients. When combining serum histone H3 levels with platelet counts, the new scoring system indicated coagulopathy with high risk of death in a simple manner. Further studies are needed to clarify the utility of serum histone H3 levels in the diagnosis of coagulopathy/DIC.

## Supplementary information

**Additional file 1: Figure S1.** ROC analyses of platelet counts and serum histone H3 levels for predicting 28-day mortality. **Table S1.** The new scoring system identified coagulopathy patients with high disease severity.

## Data Availability

The datasets used and/or analyzed during the current study are available from the corresponding author on reasonable request.

## Abbreviations

APACHE II: Acute Physiology and Chronic Health Evaluation II
AT: Antithrombin
AUC: Area under the ROC curve
DIC: Disseminated intravascular coagulation
ELISA: Enzyme-linked immunosorbent assay
FDP: Fibrin(ogen) degradation products
ICU: Intensive care unit
ISTH: International Society on Thrombosis and Haemostasis
JAAM: Japanese Association for Acute Medicine
JSTH: Japanese Society on Thrombosis and Hemostasis
PT: Prothrombin time
ROC: Receiver-operating characteristics
SIRS: Systemic inflammatory response syndrome
SOFA: Sequential Organ Failure Assessment
TAT: Thrombin-antithrombin complex
