# Supplementary material for: Serum histone H3 levels and platelet counts are potential markers for coagulopathy with high risk of death in septic patients: a single-center observational study
Source: J Intensive Care. 2019 Dec 26;7:63. doi: 10.1186/s40560-019-0420-2 (PMC6933899; doi:10.1186/s40560-019-0420-2)
Supplement: Supplementary file 1 — Additional file 1: Figure S1. ROC analyses of platelet counts and serum histone H3 levels for predicting 28-day mortality. Table S1. The new scoring system identified coagulopathy patients with high disease severity. [file 40560_2019_420_MOESM1_ESM.pdf]

Supplementary Fig. 1

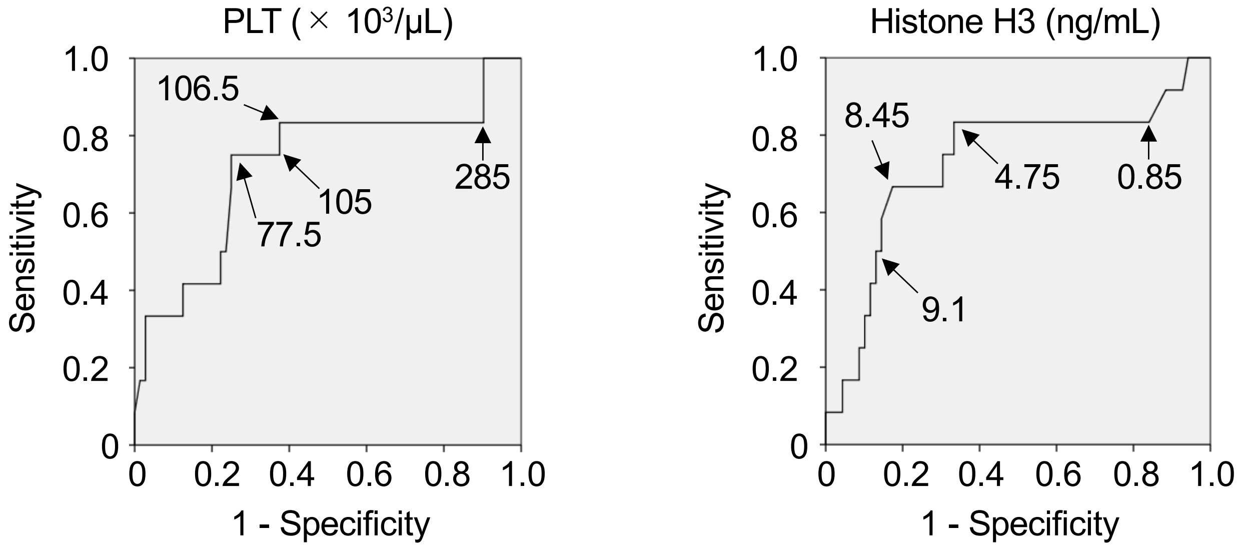


**Supplementary Fig. 1. ROC analyses of platelet counts and serum histone H3 levels for predicting 28-day mortality.** Arrows show platelet and histone H3 values around the upper left corners of the ROC curves. In ROC analysis, values corresponding to points nearest to the upper left corner of the ROC curve are considered to be good cut-off values. For predicting 28-day mortality, platelet counts of 77.5 and 106.5 × $10^3$/μL and histone H3 levels of 4.75 and 8.45 ng/mL can be good cut-off values. For predicting DIC, platelet counts of 91.5 and 136.5 × $10^3$/μL and histone H3 level of 3.55 ng/mL can be good cut-off values (data not shown). Based on these findings, the cut-off values in the new scoring system were determined to be 80 and 120 × $10^3$/μL for platelet counts and 3 and 9 ng/mL for histone H3 levels.

Supplementary Table. 1

| Number of non-survivors / patients | | DIC and SOFA score ≥ 11 | | |
|---|---|---|---|---|
| | | − | + | total |
| New criteria | − | 1 / 45 | 1 / 5 | 2 / 50 |
| | + | 3 / 9 | 6 / 12 | 9 / 21 |
| | total | 4 / 54 | 7 / 17 | 11 / 71 |

**Supplementary Table 1. The new scoring system identified coagulopathy patients with high disease severity.** The numerator and denominator of a fraction represent the number of non-survivors divided by the sum of the survivors + non-survivors, respectively, in each category. The new criteria identified 12/17 of patients with DIC and high disease severity, and 45/54 of those without. In this analysis, high disease severity was defined as a SOFA score ≥ 11 although previous studies suggested a SOFA score ≥ 13. The difference was based on the fact that our SOFA score did not include the Glasgow Coma Scale score due to invalid assessment under sedation. Similar results were obtained even if high disease severity was defined as a SOFA score ≥ 10, 11, 12, or 13. Furthermore, with respect to the remaining unmatched cohort (n = 14), our new criteria showed better prognostic performance (3/9) than the combination of DIC and SOFA scores (1/5).
